# Traumatic aortic isthmus transection with pseudoaneurysm formation and massive mediastinal hematoma: management challenges in resource-limited countries: case report

**DOI:** 10.1186/s13019-026-04382-5

**Published:** 2026-07-13

**Authors:** Tekiy M. Bedore, Ayto A. Negash, Mehreteab T. Woudineh, Dagmawi A. Teferi, Rahel G. Yitayew, Yitayew E. Mohammed

**Affiliations:** 1 Department of Emergency and Critical Care Medicine, Werabe Comprehensive Specialized Hospital, Werabe University, Werabe, Ethiopia; 2 Department of Pediatric and Child Health, St. Paul’s Hospital Millennium Medical College, Addis Ababa, Ethiopia; 3 Department of Surgery, Urology Division, St. Paul’s Hospital Millennium Medical College, Addis Ababa, Ethiopia; 4 Department of Radiology, St. Paul’s Hospital Millennium Medical College, Addis Ababa, Ethiopia; 5 Department of Internal Medicine, Werabe Comprehensive Specialized Hospital, Werabe University, Werabe, Ethiopia

**Keywords:** Blunt thoracic injury, Aortic isthmus transection, Resource-limited setting, Ethiopia, *Case Report*

## Abstract

**Background:**

Blunt thoracic aortic injury (BTAI) is rare but highly lethal, particularly at the aortic isthmus. Survival depends on early diagnosis, rapid resuscitation, and timely definitive intervention, which is challenging in resource-limited settings.

**Case presentation:**

A 35-year-old male presented 30 min after a high-impact road traffic accident in traumatic haemorrhagic shock (BP 80/50 mmHg, HR 120 bpm, RR 30/min, SpO₂ 86%). Examination revealed bilateral hemopneumothorax, rib fractures, and subcutaneous emphysema. Initial management included oxygen, bilateral chest tubes, IV fluids, and massive transfusion (5 units each of PRBC, FFP, platelets). Contrast-enhanced CT demonstrated a full-thickness aortic transection 2.5 cm distal to the left subclavian artery, forming a pseudoaneurysm with mediastinal hematoma. Despite aggressive resuscitation and attempted transfer to a tertiary vascular centre, the patient died 12 h post-injury.

**Discussion:**

Temporary survival in severe BTAI is possible with aggressive resuscitation and mediastinal tamponade. Delays in imaging, referral, and vascular expertise in resource-limited settings significantly reduce survival. Policy-level interventions (trauma networks, pre-hospital EMS) and practice-level strategies (standardized resuscitation, hypotensive protocols, protocolized transfer) are critical.

**Conclusion:**

Resuscitation can extend survival in catastrophic BTAI, but definitive surgical repair is essential. Strengthening trauma systems and referral pathways is vital in low-resource settings.

## Introduction

Blunt thoracic aortic injury (BTAI) is a rare but catastrophic consequence of high-energy trauma, including road traffic accidents, falls from height, and crush injuries [1–3]. Although it accounts for a small proportion of blunt trauma admissions, BTAI is one of the leading causes of early trauma-related mortality, second only to traumatic brain injury. Autopsy studies show that up to 80–90% of patients with BTAI die at the scene, and even those reaching hospital care face a high risk of early death [4, 5].

Historically, open thoracotomy was the standard definitive management, but it carries significant perioperative morbidity, including risks of paraplegia, stroke, and high mortality [6, 7]. Over the last two decades, thoracic endovascular aortic repair (TEVAR) has emerged as the preferred treatment in centers with vascular expertise, offering lower mortality, reduced blood loss, and fewer complications compared with open repair [8, 9].

Despite advances like TEVAR, data from low- and middle-income countries (LMICs) regarding outcomes of high-grade BTAI without timely intervention remain scarce [10–12]. This case highlights the real-world challenges faced in resource-limited settings, including delayed access to definitive care due to the absence of local vascular expertise, prolonged referral times, limited availability of advanced imaging, and lack of rapid trauma systems. Emergency resuscitation provided temporary bridging, but definitive management was delayed by 12 h, ultimately resulting in a fatal outcome.

Reporting this case addresses a critical gap in the literature by illustrating the management challenges and systemic barriers in LMICs, underscoring the need for improved trauma systems, rapid referral pathways, and access to specialized care to improve outcomes in similar settings [13–15].

## Case presentation

A 35-year-old previously healthy male was brought to the emergency department (ED) approximately 30 min after a high-impact road traffic accident. The patient had no history of chronic medical conditions and was not on any regular medications. On arrival, he was conscious but in severe distress, reporting intense chest pain and progressive shortness of breath. Initial vital signs indicated the presence of traumatic haemorrhagic shock, notable for hypotension (BP 80/50 mmHg), tachycardia (HR 120 bpm), tachypnoea (RR 30/min), and hypoxia (SpO₂ 86% on room air). Physical examination revealed reduced bilateral air entry on chest auscultation, with no obvious external bleeding or long-bone fractures.

Patient was put on supplemental oxygen via face mask, with intubation equipment prepared but not immediately required, as the patient maintained adequate spontaneous ventilation. Resuscitation was continued according to Advanced Trauma Life Support (ATLS) principles. Two large-bore intravenous lines were established, and an initial bolus of normal saline was administered. Persistent hypotension prompted transfusion of two units of whole blood, resulting in transient hemodynamic stabilization.

An extended focused assessment with sonography in trauma (eFAST) was performed within one hour of arrival, revealing bilateral hemopneumothorax and extensive subcutaneous emphysema, limiting visualization. Chest X-ray was not performed due to the patient’s critical condition and the plan for urgent thoraco-abdominal CT imaging. Bilateral tube thoracostomies were placed at the bedside, draining air with minimal blood, which temporarily improved oxygenation.

The patient remained intermittently hypotensive over the next four hours despite ongoing resuscitation, including a massive transfusion protocol (MTP). Laboratory investigations revealed normal haemoglobin and haematocrit; coagulation profile, lactate, and base excess were not available at the treating institution. A total of five units each of packed red blood cells, fresh frozen plasma, and platelets were administered during the ED stay.

**Fig. 1:**
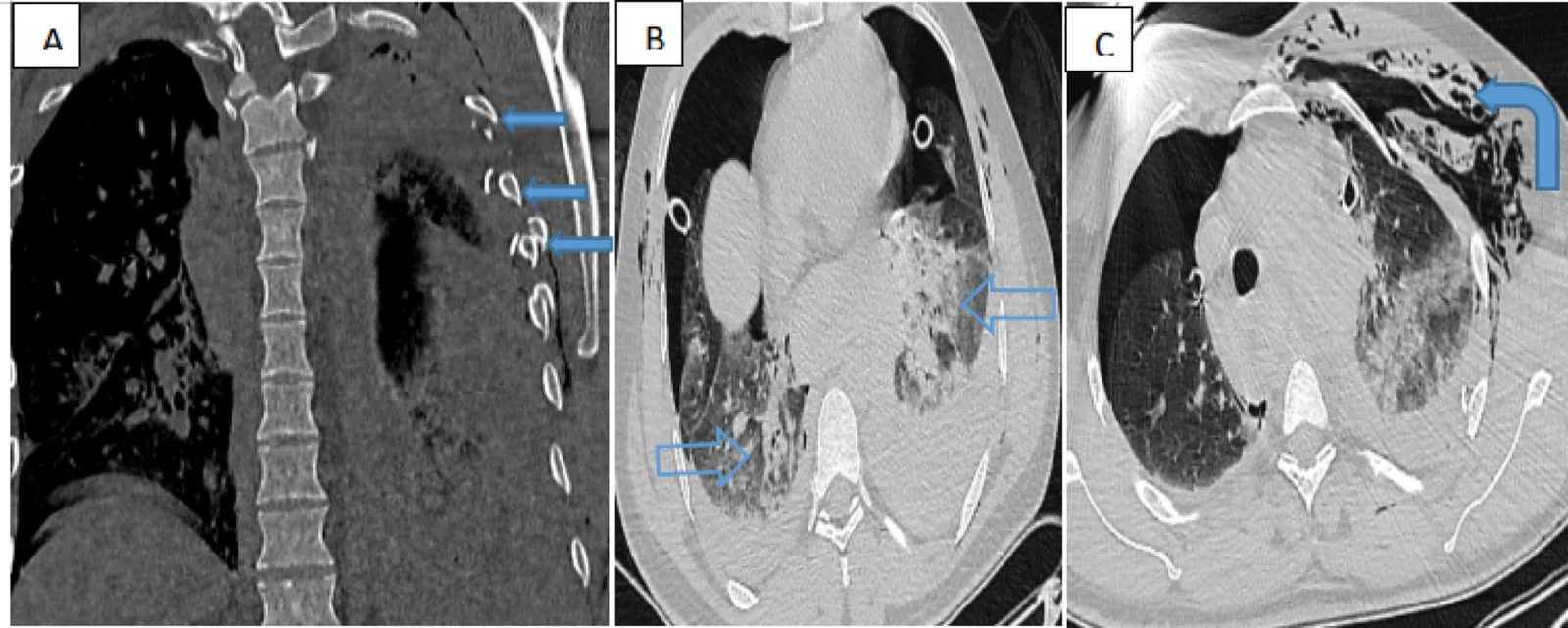
(**A**) Coronal and (**B**, **C**) Axial pre-contrast enhanced chest CT scan at bone (**A**) and chest (**B**, **C**) window. Bilateral ground-glass and consolidative opacity (straight open arrow) with bilateral near blood-attenuating pleural cavities as well as free air collection. Left 1st-7th rib fractures (straight black arrow) with mild displacement and adjacent extensive subcutaneous emphysema (curved arrow).

**Fig. 2:**
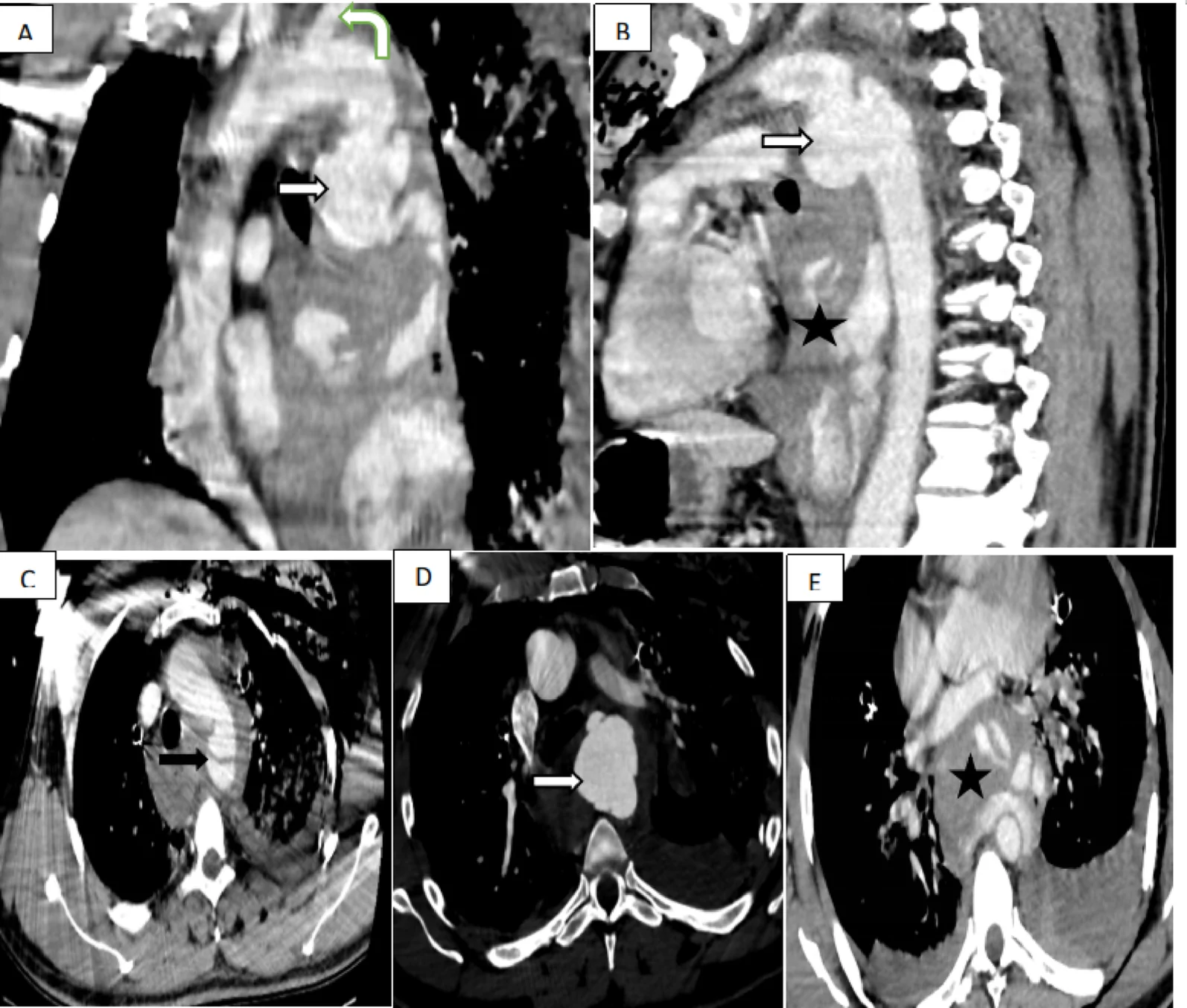
(**A**) Coronal, (**B**) Sagittal, (**C**, **D**, **E**) Axial post-contrast enhanced chest CT scan There is contour disruption of the aorta due to the full-thickness tear, with loss of continuity at its transected ends (black arrow) around the aortic isthmus, which is approximately 2.5 cm distal to the origin of the left subclavian artery (curved arrow), creating a pseudoaneurysm (white straight arrow) and surrounding hematoma extending to the mediastinum (black star), indicating rupture

To assess the full extent of injury, a contrast-enhanced thoraco-abdominal CT scan was performed four hours after arrival. Imaging revealed bilateral ground-glass opacities and consolidations consistent with lung contusion, bilateral haemothorax and pneumothorax, left 7th rib fractures, and subcutaneous emphysema (Fig. 1a–c). Aortic injury was identified in detail: post-contrast CT (Coronal, Sagittal, and Axial views; Fig. 2a–e) demonstrated a full-thickness tear of the aortic wall at the aortic isthmus, approximately 2.5 cm distal to the origin of the left subclavian artery, forming a pseudoaneurysm. Surrounding mediastinal hematoma indicated active bleeding.

Following imaging, the patient experienced persistent hypotension with poor response to transfusion. Attempts to transfer the patient to a tertiary centre with vascular surgical capabilities were initiated immediately after CT, but logistical constraints—including lack of transport and coordination with vascular teams—precluded successful transfer. At eight hours post-arrival, the patient developed cardiorespiratory arrest secondary to haemorrhagic shock. Rapid sequence intubation was performed, and mechanical ventilation was initiated.

Despite aggressive resuscitation, including advanced cardiovascular life support (ACLS), fluid therapy, and transfusions, the patient suffered cardiorespiratory arrest at 12 h post-arrival and could not be resuscitated.

## Discussion

Blunt thoracic aortic injury (BTAI) is a highly lethal form of trauma, particularly when it involves the aortic isthmus, the segment most susceptible to shear forces during rapid deceleration [8, 9, 16, 17]. Complete transection of this segment represents a grade IV injury, with extremely high mortality, up to 80–90% at the scene and over 50% within hours of hospital arrival even in resource-rich settings [2, 9, 17]. In the present case, the patient arrived alive but in profound shock, demonstrating that early hospital arrival alone does not guarantee survival without definitive surgical intervention.

Diagnosing BTAI can be challenging, especially in hemodynamically unstable patients. Initial eFAST examinations may be negative, as physical findings are often subtle or nonspecific [2, 8].

In this case, CTA was crucial for definitive diagnosis, revealing complete aortic transection and mediastinal hematoma, which guided clinical decision-making and highlighted the need for urgent transfer. Although not diagnostic for BTAI, chest X-rays remain a rapid, widely available screening tool and can reveal indirect signs such as mediastinal widening, apical cap, pleural effusion, or hemothorax, which should prompt suspicion for aortic injury [18, 19].

These interventions provided a “resuscitative window,” allowing temporary hemodynamic stability in the absence of definitive repair. Hypotensive resuscitation, aiming to maintain systolic blood pressure sufficient for end-organ perfusion but low enough to reduce stress on the aortic wall, may delay rupture in such cases [20, 21]. Additionally, mediastinal hematoma likely tamponade the aortic rupture, further prolonging survival.

Bilateral chest tube insertion improved oxygenation by decompressing hemopneumothoraxes. However, sudden changes in intrathoracic pressure can influence aortic wall stress. Rapid decompression may increase transmural pressure across the aorta, potentially accelerating rupture, while controlled decompression could mitigate this risk. Awareness of these physiologic dynamics is important in managing high-grade BTAI in resource-limited settings.

Despite ongoing resuscitation, the patient developed worsening acidosis and ongoing hypotension. Coagulopathy and metabolic derangements likely contributed to continued instability, reflecting the importance of timely definitive intervention. Continuous correction of acidosis, balanced transfusion strategies, and vigilant monitoring are essential components of bridging management to sustain temporary survival.

This case highlights systemic barriers in rural Ethiopia, including lack of local vascular expertise, limited imaging and delayed interpretation, poorly coordinated referrals, and constrained transport to tertiary centers. These factors prolong time to definitive care and critically reduce survival in catastrophic thoracic vascular injuries.

These factors collectively prevented timely definitive management and contributed to the fatal outcome. Similar challenges have been reported in other low- and middle-income countries, emphasizing the global disparity in trauma care [2, 22–24].

**Policy-level interventions** should focus on strengthening national trauma systems and improving access to definitive care. Establishing standardized trauma referral pathways and regional networks can facilitate rapid transfer of patients with catastrophic vascular injuries. Investment in training programs for vascular and trauma surgery, including endovascular techniques, at regional centers is essential to build local capacity. Additionally, the development of pre-hospital emergency medical services with efficient triage and transport capabilities can ensure timely access to specialized care.

**Practice-level strategies** should emphasize standardized and coordinated patient management. Protocols for initial resuscitation, monitoring, and stabilization prior to transfer can optimize patient condition during delays. Telemedicine or mobile vascular teams can provide real-time guidance to peripheral facilities lacking specialized expertise. The use of hypotensive resuscitation and massive transfusion protocols can help extend survival until definitive treatment is available. Finally, implementing structured secondary transport protocols—including pre-transfer stabilization, oxygenation, and circulatory support—can improve patient safety during transfer to tertiary centers.

Survival beyond a few hours after complete aortic transection is extremely rare worldwide. Case series and registry studies report that most patients die at the scene or within the first 2–6 h of injury [2–25]. Temporary survival beyond 12 h, as demonstrated in this case, is unusual and highlights the potential value of aggressive, structured resuscitation to extend the window for definitive intervention.

This report has several limitations. First, high-quality imaging could not be obtained due to equipment constraints; only the available CT images are included. Second, certain laboratory parameters, including lactate, coagulation profile, and base excess, were not available, limiting a full assessment of physiological derangements. Third, as a single-case report, findings cannot be generalized, and systemic recommendations are based on extrapolation rather than direct evidence from multiple cases.

The case emphasizes that survival in high-grade BTAI depends not only on hospital care but also on system-level preparedness. Even with optimal resuscitation, delayed definitive repair can be fatal. Investment in trauma networks, rapid diagnostic protocols, pre-hospital services, and vascular/endovascular capacity is essential to improve outcomes in LMICs. While a single case cannot provide definitive evidence for systemic interventions, it provides valuable lessons regarding the importance of bridging strategies, rapid stabilization, and coordinated referral pathways.

## Conclusion

This case demonstrates that temporary survival after complete aortic transection is possible with aggressive resuscitative measures, even in resource-limited settings. However, systemic delays in definitive care can negate these efforts. Reporting such cases informs global trauma practice, highlights survivable windows, and underscores the need for strengthened trauma systems, rapid referral pathways, and capacity-building in vascular and endovascular care.

Learning Points

- High lethality of aortic isthmus transection: - Survival to hospital presentation is rare, and definitive repair is essential for survival.
- Temporary survival with aggressive resuscitation: - Short-term stabilization is possible with massive transfusion and vasopressor support, even without immediate surgery.
- Challenges in resource-limited settings: - Lack of access to specialized vascular care significantly impacts survival outcomes.
- Need for improved trauma systems and referral pathways: - Strengthening these systems can improve outcomes for patients with catastrophic thoracic vascular injuries.

## Data Availability

No datasets were generated or analysed during the current study.

## Abbreviations

BTAI: Blunt thoracic aortic injury
eFAST: Extended focused assessment with sonography for trauma
GCS: Glasgow coma scale
ROSC: Return of spontaneous circulation
TEVAR: Thoracic endovascular aortic repair
MPT: Massive transfusion protocol
POCUS: Point-of-care ultrasound
CT: Computed tomography
ACLS: Advanced cardiac life support

## Declarations

### Duplicate publication

The authors confirm that this manuscript has not been published previously, is not under consideration for publication elsewhere, and will not be submitted elsewhere while under review by BMC Cardiovascular Disorders. All authors approve its submission. Artificial Intelligence: No artificial intelligence tools or large language models were used in the writing, analysis, or generation of this manuscript. All content was produced by the authors.
